# Soluble Klotho: a possible predictor of quality of life in acromegaly patients

**DOI:** 10.1007/s12020-020-02306-4

**Published:** 2020-04-24

**Authors:** Eva C. Coopmans, Nour El-Sayed, Jan Frystyk, Nils E. Magnusson, Jens O. L. Jørgensen, Aart-Jan van der Lely, Joop A. M. J. L. Janssen, Ammar Muhammad, Sebastian J. C. M. M. Neggers

**Affiliations:** 1 Department of Medicine, Section Endocrinology, Pituitary Center Rotterdam, Erasmus University Medical Center, Rotterdam, The Netherlands; 2 Medical Research Laboratory, Department of Clinical Medicine, Aarhus University, Aarhus, Denmark; 3 Department of Clinical Research, Faculty of Health Sciences, University of Southern Denmark, Odense, Denmark; 4 Department of Endocrinology, Odense University Hospital, Odense, Denmark; 5 Department of Endocrinology and Internal Medicine, Aarhus University Hospital, Aarhus, Denmark

**Keywords:** Acromegaly, Alpha Klotho, QoL, Active disease, Somatostatin receptor ligand

## Abstract

**Purpose:**

Although quality of life (QoL) is improved in patients with acromegaly after disease control, QoL correlates only weakly with traditional biomarkers. Our objective is to investigate a potential relation between the new serum biomarker soluble Klotho (sKlotho), GH and insulin-like growth factor 1 (IGF-1) levels, and QoL.

**Methods:**

In this prospective cohort study, we investigated 54 acromegaly patients biochemically well-controlled on combination treatment with first-generation somatostatin receptor ligands (SRLs) and pegvisomant (PEGV) at baseline and 9 months after switching to pasireotide LAR (PAS-LAR; either as monotherapy, *n* = 28; or in combination with PEGV, *n* = 26). QoL was measured by the Patient-Assessed Acromegaly Symptom Questionnaire (PASQ) and Acromegaly Quality of Life (AcroQoL) questionnaire.

**Results:**

Switching to PAS-LAR treatment significantly improved QoL without altering IGF-1 levels. QoL did not correlate with GH or IGF-1 levels, but sKlotho correlated with the observed improvements in QoL by the AcroQoL global (*r* = −0.35, *p* = 0.012) and physical subdimension (*r* = −0.34, *p* = 0.017), and with PASQ headache (*r* = 0.28, *p* = 0.048), osteoarthralgia (*r* = 0.46, *p* = 0.00080) and soft tissue swelling score (*r* = 0.29, *p* = 0.041). Parallel changes in serum sKlotho and IGF-1 (*r* = 0.31, *p* = 0.023) suggest sKlotho and IGF-1 to be similarly dependent on GH. Comparing the PAS-LAR combination therapy and the monotherapy group we did not observe a significant difference in improvement of QoL.

**Conclusions:**

Patients experienced improved QoL during PAS-LAR, either as monotherapy or in combination with PEGV. Soluble Klotho concentrations appear to be a useful marker of QoL in acromegaly patients but the underlying mechanisms remain to be investigated.

## Introduction

Medical treatment of acromegaly with the combination of the second-generation somatostatin receptor ligand (SRL) pasireotide long-acting release (PAS-LAR) and the GH receptor antagonist, pegvisomant (PEGV) provides control of insulin-like growth factor I (IGF-1) levels in most (77.0%) patients [1, 2]. To this date, GH and especially GH activity, as reflected (in part) by IGF-1 concentrations in serum, are considered the ‘classical’ biomarkers to diagnose acromegaly and monitor disease activity during treatment. Normalization of GH and IGF-1 levels has been associated with normalization in mortality rates [3–6], while not necessarily reflecting optimal quality of life (QoL) in acromegalic patients [7–11].

The *Klotho* gene was originally identified as an ageing-suppressor gene of restricted expression (predominantly in the kidney, brain, and parathyroid and pituitary glands), encoding a transmembrane protein, mKlotho. The extracellular domain of mKlotho is found as a circulating soluble α-Klotho (sKlotho) into blood, cerebrospinal fluid, and urine [12, 13]. Recent data suggest that sKlotho levels are elevated in patients with active acromegaly [14, 15]. After surgical removal of the GH-producing pituitary adenoma alone or in combination with first-generation SRLs they decrease toward the normal range [14–17]. Overall, when assessing patients with acromegaly, concomitant and parallel changes in serum sKlotho and IGF-1 were observed over time in each patient, and levels of sKlotho and IGF-1 appeared to be similarly dependent on GH [14, 18]. However, the mechanisms by which acromegaly leads to excess sKlotho remain to be elucidated. Further, sKlotho has been proposed to inhibit IGF-1 and insulin receptor signaling by inhibiting tyrosine phosphorylation of both receptors and their downstream signaling proteins (i.e., IRS) [19–21]. Thus, sKlotho seems to be a new player in the intricate regulation of the GH and IGF-1 axis. As sKlotho concentrations appear to reflect acromegaly disease activity [14, 15], it may potentially serve as a more integrated serum biomarker of disease-specific QoL.

Normalized GH and IGF-1 levels do not always coincide with symptom relief [11], which may be explained by ‘extra-hepatic acromegaly’ [22]. In addition to suppression of GH secretion from the pituitary tumor, SRLs also suppress insulin secretion in the portal vein, which by itself downregulates hepatic IGF-1 production via GH receptors. However, the GH action in the peripheral tissues remains unaltered and might still have acromegaly-inducing effects [22]. In other words, integrated extra-hepatic GH activity may remain elevated despite normalized serum IGF-1 levels in these patients. If these extra-hepatic GH actions could be antagonized by the addition of PEGV in patients using first- or second-generation SRLs, one might observe an improvement of QoL in comparison with SRL monotherapy. In fact, it has been shown previously that the addition of PEGV to first-generation long-acting SRL therapy can improve GH-dependent parameters of QoL [23]. To date, however, there is no data about second-generation SRL (i.e., PAS-LAR) and PEGV combination therapy and its effect on QoL nor a convenient biomarker of extra-hepatic disease activity in acromegaly.

We performed a prospective intervention study in well-controlled acromegaly patients on first-generation SRLs and PEGV combination therapy. Soluble Klotho, GH and IGF-1 levels, and QoL were assessed before and after switching to PAS-LAR alone or PAS-LAR and PEGV combination therapy for a period of 9 months. The rationale for initiating the combination treatment is based on the concept that the addition of PEGV could antagonize the so-called extra-hepatic GH actions, and thereby improve QoL in comparison with PAS-LAR monotherapy. Aims of the study are: (1) to investigate the value of sKlotho in monitoring QoL; (2) and to establish the QoL during PAS-LAR treatment, and whether changes in disease activity can be differentiated in the PAS-LAR monotherapy and combination therapy group.

## Materials and methods

### Patients and study design

**Fig. 1:**
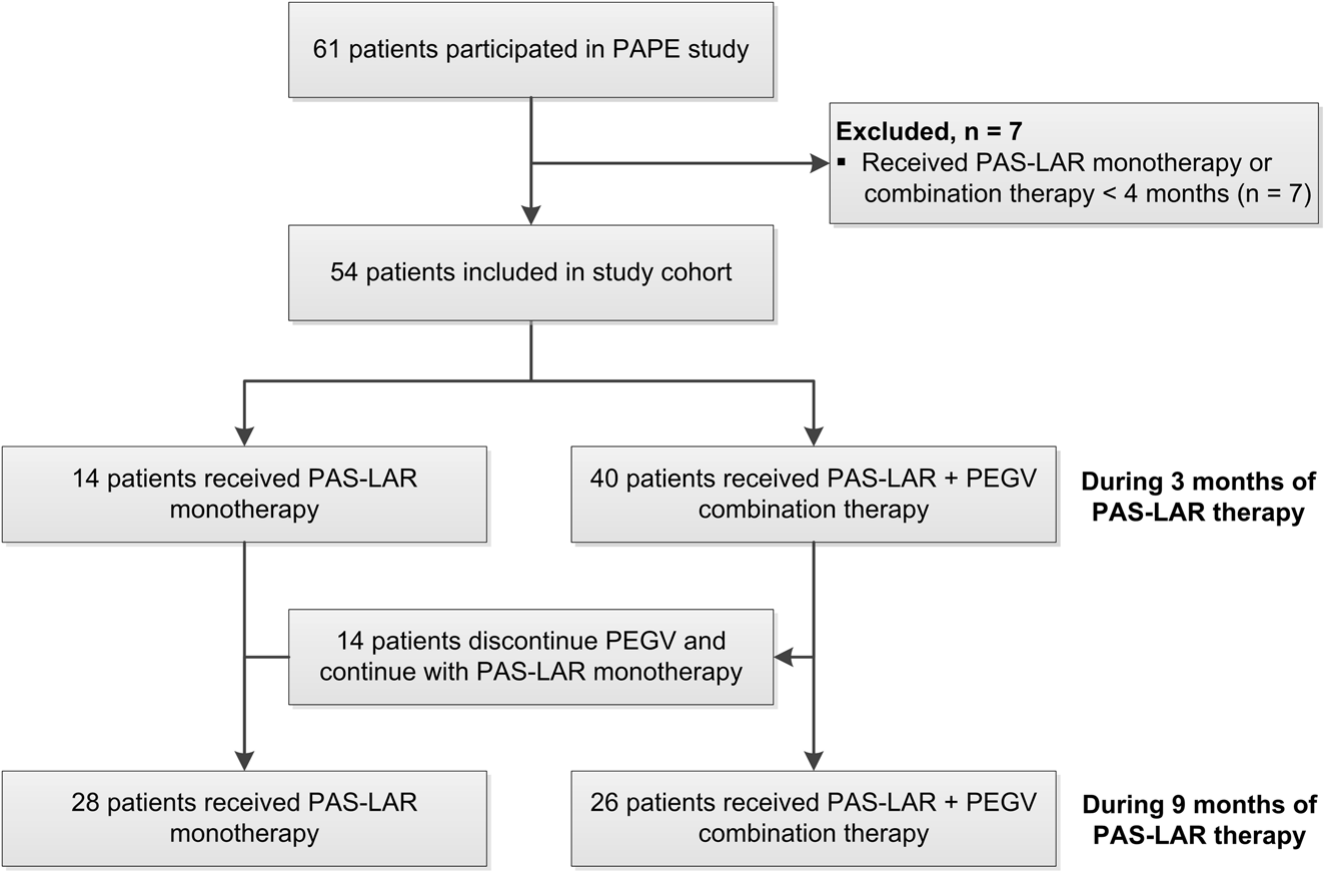
Flowchart of the selection procedure for the study cohort. All patients eventually received first-generation SRL and PEGV combination treatment and were switched to pasireotide LAR treatment during the PAPE study. PAPE pasireotide LAR and pegvisomant study, PAS-LAR pasireotide long-acting release, PEGV pegvisomant, SRL somatostatin receptor ligand

Data collection of acromegaly patients was performed at the outpatient clinic of the Pituitary Center Rotterdam, Erasmus University Medical Center in Rotterdam. We initially started with a cohort of 61 patients who received PAS-LAR treatment during their participation in the PAPE study (Fig. 1); details of the study design have been reported previously [1, 2]. Patients who received either PAS-LAR monotherapy or combination therapy less than 4 months were excluded (7 of the 61, Fig. 1). Briefly, all patients were previously treated with first-generation SRLs, followed by PEGV and first-generation SRL combination therapy. At baseline, the PEGV dose was reduced by 50% up to 3 months. When IGF-1 remained ≤1.2 × ULN after 3 months, patients were switched to PAS-LAR 60 mg monotherapy for 3 months. When IGF-1 was >1.2 × ULN, patients were switched to PAS-LAR 60 mg, and they continued with the 50% reduced PEGV dose for three months. During the extension phase until 9 months of PAS-LAR treatment, the goal was to achieve IGF-1 normalization (IGF-1 ≤ 1.2 × ULN) through protocol-based dose titration of PEGV and PAS-LAR [1, 2].

We prospectively collected data on PAS-LAR, while data on medical history and clinical response to patients during first-generation SRLs treatment were retrospectively collected.

### Blood measurements

Total IGF-1 concentrations were measured by chemiluminescent immunometric assay (IDS-iSYS; Immunodiagnostic Systems, Boldon, United Kingdom) and were interpreted according to the age- and sex-dependent ranges [24]. GH levels were measured using the IDS-iSYS assay, which is free of interference from PEGV [25]. Soluble α-Klotho levels were assessed using a solid phase sandwich ELISA described by Yamazaki et al. [26] (IBL; IBL International GmbH, Hamburg, Germany) according to the manufacturer’s instructions. Absolute changes in IGF-1, GH, and sKlotho were calculated by subtracting follow-up concentrations from concentrations at baseline.

### QoL questionnaires

The Patient-Assessed Acromegaly Symptom Questionnaire (PASQ) and Acromegaly Quality of Life (AcroQoL) questionnaire were used to evaluate symptoms and QoL at baseline and after 9 months of PAS-LAR treatment [27–30].

The PASQ is a disease-specific questionnaire, which consists of six questions scoring 0–8 [31]. These questions evaluate symptoms such as headache, excessive perspiration, osteoarthralgia, fatigue, soft tissue swelling, and paresthesia. The seventh question addresses the overall health status, based on the other six questions, scoring 0–10. The maximum score is 48 and indicates severe signs and symptoms.

The AcroQoL questionnaire comprises 22 questions, with each having five possible answers scoring 1–5. The questions are divided into two main categories: physical and psychological function; the latter is subdivided into appearance and personal relationships [27, 29]. The score of 110 (100%) reflects the best possible AcroQoL score. The AcroQoL questionnaire has a good internal consistency (Cronbach’s α > 0.7) [30].

### Statistical analysis

For the analysis of QoL questionnaires, changes between baseline and during 9 months of PAS-LAR treatment were calculated (changes in QoL; i.e., subtracting follow-up from baseline scores). To assess improvement in QoL scores during the study, we used the minimally important difference (MID) to define improvement in QoL scores between baseline and 9 months of PAS-LAR. MID is defined as an improvement of >50% of the baseline standard deviation (SD) of the baseline mean QoL score [28].

For missing data, imputation based on last observation carried forward was used. Continuous data were expressed as mean and SD or median and interquartile range (IQR), as appropriate. Categorical data were represented as observed frequencies and percentages. The Kolmogorov–Smirnov test was used to determine normality of variables. Logarithmic transformations were applied to variables that did not meet the criteria of normality. We compared categorical variables between the two groups with the *χ*^2^ test, continuous variables between groups with either the Student’s *t* test or the Mann–Whitney *U* test and either the paired Student’s *t* test or the Wilcoxon signed rank test for two related groups. Correlation analyses were performed using Spearman’s rank-correlation coefficient. *P* < 0.05 was considered statistically significant without any multiplicity correction. Statistical analyses were performed using SPSS software (version 24 for Windows; SPSS Inc., Chicago, IL) and GraphPad Prism® version 6.04 (GraphPad Software, San Diego, CA).

## Results

### Clinical characteristics of study population

All 54 patients completed the study. Cohort demographics and clinical characteristics are summarized in Table 1. After 3 months, 14 (25.9%) of 54 patients were on PAS-LAR 60 mg monotherapy every 4 weeks, increasing to 28 (51.9%) patients during 9 months of PAS-LAR treatment (see Fig. 1 and Supplementary Fig. 1). The remaining 40 (74.1%) patients with elevated IGF-1 levels continued with their reduced dose of PEGV treatment, but now in combination with PAS-LAR 60 mg, decreasing to 26 (48.1%) patients during 9 months of PAS-LAR treatment At baseline, the PEGV dose was significantly higher in the PAS-LAR combination therapy group compared with the PAS-LAR monotherapy group (median 160 mg/week [IQR 100–225] vs. 60 mg/week [60–80], *p* ≤ 0.0001). In the PAS-LAR combination therapy group, the average total reduction in PEGV dose was 38.1% (median 80 mg/week [IQR 48–155] vs. 160 mg/week [100–225], *p* = 0.0031) after 9 months of PAS-LAR treatment.

**Table 1:** Cohort demographics and clinical characteristics of the total group, PAS-LAR monotherapy and combination therapy subgroup

Clinical characteristics	All patients *n* = 54	PAS-LAR monotherapy *n* = 28	PAS-LAR and PEGV combination therapy *n* = 26
Age (years)	53.3 (11.9)	56.4 (11.7)	50.0 (11.3)*
Female patients	26 (48.1%)	15 (53.6%)	11 (42.3%)
Time since diagnosis (years)	8.5 (5.0–13.0)	10.5 (6.3–15.0)	7.0 (2.0–11.0)*
Previous treatment			
Surgery	25 (46.3%)	16 (57.1%)	9 (34.6%)
Surgery and radiotherapy	6 (11.1%)	4 (14.3%)	2 (7.7%)
Primary medical therapy	29 (53.7%)	12 (42.9%)	17 (65.4%)
Pituitary insufficiency			
Panhypopituitarism	3 (5.6%)	2 (7.1%)	1 (3.8%)
1–2 axes	25 (46.3%)	12 (42.9%)	13 (50.0%)
No hypopituitarism	26 (48.2%)	14 (50.0%)	12 (46.2%)
Duration of SRL + PEGV treatment (years)	5.9 (3.4–8.9)	7.0 (4.8–9.2)	4.5 (1.6–8.1)
PEGV dose (mg/week) at baseline	80 (60–160)	60 (60–80)	160 (100–225)*
Presence of diabetes during PAS-LAR	38 (70.4%)	20 (71.4%)	18 (69.2%)
Insulin therapy during PAS-LAR	3 (5.6%)	1 (3.6%)	2 (7.7%)

Data are mean (SD), median (IQR) or number (%)

Asterisk represents *p* ≤ 0.05 for the comparisons between PAS-LAR monotherapy and PAS-LAR and PEGV combination therapy and are derived from the Student’s *t* test (continuous variables) and Pearson’s *χ*^2^ test (categorical variables)

*IGF-*1 insulin-like growth factor 1, *PAS-LAR* pasireotide long-acting release, *PEGV* pegvisomant, *SRL* somatostatin receptor ligand

At baseline, age and time since diagnosis of the patients receiving PAS-LAR monotherapy were significantly higher compared with the combination therapy group (age, mean 56.4 years [SD 11.7] vs. 50.0 years [11.3], *p* = 0.047; time since diagnosis, median 10.5 years [IQR 6.3–15.0] vs. 7.0 years [2.0–11.0], *p* = 0.020).

### Baseline sKlotho, GH and IGF-1 axis

**Fig. 2:**
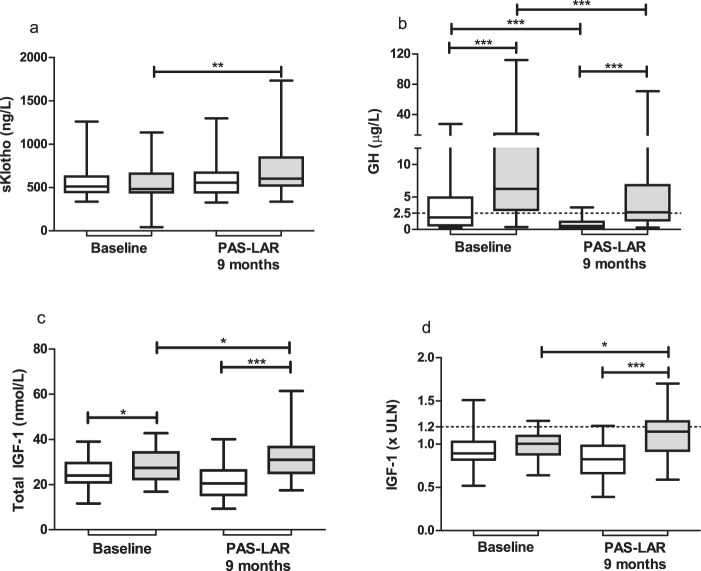
Soluble Klotho (**a**) levels and random GH (**b**) at baseline and during PAS-LAR monotherapy or in combination with PEGV. IGF-1 (**c**) and IGF-1 (x ULN) (**d**) levels at baseline and during PAS-LAR monotherapy or in combination with PEGV. The white boxes represent the PAS-LAR monotherapy group, while the gray boxes represent the PAS-LAR and PEGV combination therapy group. Box whisker plots are expressed in minimum, median, and maximum. Insulin-like growth factor 1 IGF-1, PAS-LAR pasireotide long-acting release, sKlotho soluble Klotho, ULN upper limit of normal

At baseline, serum sKlotho levels were not significantly different between patients receiving 9 months of PAS-LAR mono- versus patients using PAS-LAR and PEGV combination therapy (median 513 ng/L [IQR 445–627] vs. 482 [443–660], *p* = 0.44, Fig. 2a). GH and total IGF-1 levels at baseline were significantly higher in the patients who did receive combination therapy during the next 9 months when compared with patients who did receive PAS-LAR monotherapy during the next 9 months (GH, median 6.3 µg/L [IQR 3.0–14.5] vs. 1.9 [0.6–5.0], *p* = 0.00030, Fig. 2b; total IGF-1, median 27.4 nmol/L [IQR 22.4–34.3] vs. 24.0 [20.9–29.6], *p* = 0.038; Fig. 2c). Serum IGF-1 (x upper limit of normal (ULN)) levels at baseline, however, were not significantly different between the two groups (median 0.90 [IQR 0.8–1.0] vs. 1.0 [0.9–1.1], *p* = 0.27; Fig. 2d) and were within the age-adjusted normal range.

### Follow-up sKlotho, GH and IGF-1 axis

When comparing follow-up to baseline, we observed a significant increase in sKlotho levels in the combination therapy group (median 482 ng/L [IQR 443–660] vs. 603 [521–847], *p* = 0.0016, Fig. 2a), but no significant change in the monotherapy group (513 [445–627] vs. 555.0 [440–671], *p* = 0.30, Fig. 2a). During follow-up, sKlotho levels were not different between the two treatment groups. Due to the PEGV dose reduction or discontinuation during the study, GH levels were significantly lower in both groups when compared with baseline (monotherapy, median 1.9 µg/L [IQR 0.6–5.0] vs. 0.6 [0.3–1.2], *p* = 0.0082; combination therapy, 6.3 [3.0–14.5] vs. 2.7 [1.4–6.8], *p* = 0.0048, Fig. 2b). Total and age-dependent serum IGF-1 levels showed a significant increase between baseline and follow-up in the combination therapy group (total IGF-1, median 27.4 nmol/L [IQR 22.4–34.3] vs. 31.0 [25.1–36.7], *p* = 0.016; IGF-1 (x ULN), median 1.0 [IQR 0.9–1.1] vs. 1.1 [0.9–1.3], *p* = 0.027, respectively Fig. 2c, d). No changes in serum IGF-1 were seen in the monotherapy group.

As previously published by Muhammad et al. comparing follow-up to baseline, total and age-dependent serum IGF-1 levels, as well as GH levels were significantly higher in patients treated with combination therapy compared with patients treated with monotherapy (total IGF-1, median 31.0 nmol/L [IQR 25.1–36.7] vs. 20.5 [15.4–26.3], *p* ≤ 0.0001; IGF-1 (x ULN), median 1.1 [IQR 0.9–1.3] vs. 0.8 [0.7–1.0], *p* ≤ 0.0001, respectively Fig. 2c, d; GH, median 2.7 µg/L [IQR 1.4–6.8] vs. 0.6 [0.3–1.2], *p* = 0.0085, Fig. 2b) [2].

### Baseline QoL

The PASQ and AcroQoL scores of the total cohort at baseline and follow-up are summarized in Table 2. At baseline, the most severe symptoms of the PASQ were osteoarthralgia and fatigue, whereas excessive perspiration, soft tissue swelling, paresthesia, and headache were the least burdensome. With regards to the AcroQoL score, the greatest impairments were detected in the physical and appearance subdimensions, while the personal relations and total psychological subdimensions were the least impaired. AcroQoL and PASQ scores were not significantly different between the PAS-LAR mono- and combination therapy group at baseline (Table 2).

**Table 2:** Quantitative changes in QoL induced by the switch to PAS-LAR monotherapy and in combination with PEGV combined

	Baseline median (IQR) *n* = 51	PAS-LAR (9 months) median (IQR) *n* = 51	*P* values
PASQ total	14.0 (7.0–21.3)	10.0 (6.0–19.0)	0.090
PASQ headache	1.0 (0.0–3.0)	1.0 (0.0–2.0)	0.014
PASQ excessive perspiration	2.0 (0.0–3.8)	2.0 (0.0–4.0)	ns
PASQ osteoarthralgia	3.5 (1.0–5.0)	3.0 (1.5–4.5)	ns
PASQ fatigue	3.0 (2.0–5.0)	3.0 (1.5–5.0)	0.0074
PASQ soft tissue swelling	2.0 (0.0–3.0)	1.0 (0.0–3.0)	ns
PASQ paresthesia	1.0 (0.0–3.0)	1.0 (0.0–3.0)	ns
PASQ overall health status	3.0 (2.0–6.0)	3.0 (2.0–5.0)	ns
AcroQoL global (%)	66.4 (60.0–77.3)	71.8 (65.0–82.7)	≤0.0001
AcroQoL physical (%)	31.4 (25.7–35.7)	35.7 (29.3–40.0)	≤0.0001
AcroQoL psychological (%)	70.7 (64.3–78.6)	72.9 (65.0–85.7)	0.0051
AcroQoL personal relations (%)	80.0 (68.6–88.6)	82.9 (71.4–88.6)	ns
AcroQoL appearance (%)	62.9 (54.3–77.1)	65.7 (54.3–77.1)	0.0095

*AcroQoL* Acromegaly Quality of Life, *IQR* interquartile range, *ns* not significant, *PAS-LAR* pasireotide long-acting release, *PASQ* Patient-Assessed Acromegaly Symptom Questionnaire, PEGV pegvisomant

### Follow-up QoL

Significant differences in the total cohort between baseline and during 9 months of PAS-LAR treatment were detected in the individual PASQ scores for headache (Δ0, *p* = 0.014, MID 23.5%) and fatigue (Δ0, *p* = 0.0074, MID 25.5%). Improvements in the total PASQ score were of borderline significance only (Δ − 4.0, *p* = 0.090) with a MID of 33.3% of patients during 9 months of PAS-LAR compared with baseline. Significant improvements were observed in all AcroQoL subdimensions; physical (Δ4.3, *p* ≤ 0.0001), psychological (Δ2.2, *p* = 0.0051), and appearance (Δ2.8, *p* = 0.0095), except for personal relations. The greatest significant improvement was observed in the physical subdimension, from 31.4% at baseline to 35.7% at 9 months (Δ4.3, *p* ≤ 0.0001) with a MID of 54.9% of patients. The AcroQoL global score also improved significantly after treatment with PAS-LAR during the study (Δ5.4, *p* ≤ 0.0001). In fact, 47.1% of the patients experienced a MID.

### Treatment group stratification

When stratifying the total cohort by therapy group (PAS-LAR mono- and combination therapy), AcroQoL and PASQ scores were not significantly different between the PAS-LAR mono- and combination therapy group at baseline or follow-up (data not shown). However, a MID in soft tissue swelling during PAS-LAR treatment tended to present more frequently in the combination group compared with the monotherapy group (ten patients (50.0%) vs. five patients (22.7%), *χ*^2^*p* = 0.065). The total and other individual PASQ scores did not differ significantly between the groups after treatment with PAS-LAR. Similarly, neither the AcroQoL global nor any of the subdimensions differed significantly between the combination and monotherapy group after treatment with PAS-LAR. AcroQoL and PASQ scores in the treatment groups were comparable with the total cohort, with exception of the change in fatigue, which only presented in the combination group (not shown).

### Correlations

**Fig. 3:**
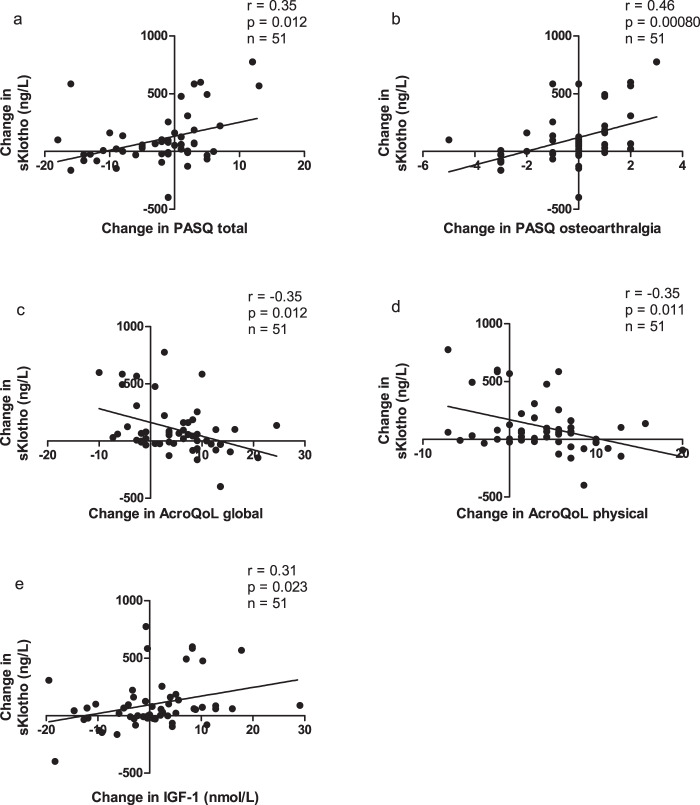
Correlations between the change in sKlotho and PASQ total (**a**) and PASQ osteoarthralgia (**b**). Correlations between the change in sKlotho and AcroQoL global (**c**) and AcroQoL physical dimension (**d**). **e** Depicts the correlation between change in sKlotho and change in total IGF-1 levels adjusted for age. AcroQoL Acromegaly Quality of Life, IGF-1 insulin-like growth factor 1, sKlotho soluble Klotho, PASQ Patient-Assessed Acromegaly Symptom Questionnaire

The correlations between changes in parameters of QoL and changes in biochemical parameters in the total cohort are presented in Fig. 3. Neither the change in IGF-1 nor the baseline and/or change in GH levels correlated with changes in QoL in the total cohort (not shown). In contrast, changes in sKlotho levels correlated with PASQ total (*r* = 0.35, *p* = 0.012, Fig. 3a), headache (*r* = 0.28, *p* = 0.048), osteoarthralgia (*r* = 0.46, *p* = 0.00080, Fig. 3b), and soft tissue swelling score (0.29, *p* = 0.041), which are all QoL entities that reflect increased GH actions. In addition, changes in sKlotho levels correlated with the observed improvements in QoL by the AcroQoL global (*r* = −0.35, *p* = 0.012, Fig. 3c) and the physical subdimension (*r* = −0.34, *p* = 0.017, Fig. 3d). PASQ fatigue (*r* = 0.23, *p* = 0.10) and excessive perspiration (*r* = 0.24, *p* = 0.091), and the AcroQoL personal relations subdimension (*r* = −0.26, *p* = 0.061) all tended to correlate with changes in sKlotho levels but failed to reach significance. At last, changes in sKlotho were positively correlated with changes in total IGF-1 levels (*r* = 0.31, *p* = 0.023, adjusted for age, Fig. 3e).

### Soluble Klotho and reasons to continue PAS-LAR therapy

At the end of study, 61.1% (33/54) patients chose to continue PAS-LAR treatment with or without PEGV, instead of switching back to their previous first-generation SRL and PEGV combination treatment regimen. The two most frequently reported reasons for continuation with PAS-LAR were the improvement in fatigue and headache symptoms (20/33, 60.6%), followed by the achieved PEGV dose reduction or discontinuation (13/33, 39.4%). The most frequently reported reason for discontinuing PAS-LAR was the use of antidiabetic medications (17/21, 81.0%) followed by experiencing no improvement in QoL (3/21, 14.3%) or newly occurring ECG abnormalities (1/21, 4.7%).

All patients were stratified according to their decision on whether to continue or discontinue PAS-LAR treatment and subsequently compared with sKlotho concentrations. Changes in sKlotho levels were inversely correlated with the achieved PEGV dose reduction and discontinuation during 9 months of PAS-LAR treatment (*r* = −0.38, *p* = 0.0049). In addition, the achieved PEGV dose reduction tended to correlate with sKlotho levels during 9 months of PAS-LAR treatment (*r* = −0.24, *p* = 0.089) but failed to reach significance.

## Discussion

The most striking finding of our study was that serum sKlotho correlates with QoL, while GH and IGF-1 do not. In our study the improvement of QoL correlated with the improvement in serum sKlotho (i.e., a greater absolute reduction in sKlotho). This is line with previous data suggesting that sKlotho levels are elevated in patients with active acromegaly and decrease toward the normal range after surgery [14, 15] and/or first-generation SRLs [16, 17].This suggests that sKlotho might be a potentially useful biomarker in assessing QoL in acromegaly patients. Furthermore, we observed that improvement in serum sKlotho corresponds with improvement in serum IGF-1. This is in line with the previous study of Anand et al. that showed that IGF-1 and sKlotho levels decrease to a similar extent during surgery alone or in combination with medical treatment [17]. Thus, it may also provide clinicians with another tool, apart from GH and IGF-1, to assess disease activity in acromegaly. This observation confirms and builds upon previous studies [12, 14, 15], proposing sKlotho to be an independent marker of disease severity in acromegaly besides GH and IGF-1. It has previously been stated that serum GH and IGF-1 levels, assessed by the commercially available assays, do not correlate well enough with the patients’ perception of QoL to use them for defining proper biochemical control [7–11, 23]. Interestingly, although the disease-specific osteoarthralgia and soft tissue swelling score remained unimproved during 9 months with PAS-LAR, the symptoms were significantly correlated to sKlotho but not to IGF-1 or GH.

Our results are in contrast to a previous study by Varewijck et al., which failed to detect significant relations between sKlotho and any of the QoL measurements in treatment-naive acromegaly patients, while both studies employed the same assay to assess sKlotho [32]. However, only one sKlotho measurement was performed in the study of Varewijck et al. and the small number of included patients (*n* = 15) may have increased the risk for a type 2 error [32]. Indeed, in the present study we measured sKlotho during first-generation SRL and PEGV combination treatment at baseline and after switching to PAS-LAR treatment alone or in combination with PEGV. In contrast to naive acromegaly patients, the patients included in the present study may show long-term effects of treatment, such as postirradiation effects or hypopituitarism.

Soluble Klotho concentrations appear to be a useful marker of QoL in acromegaly patients, but the underlying mechanisms have to be investigated. Although our data suggest a predominant role for sKlotho in the intricate regulation of the GH-IGF-1 axis, the underlying mechanisms whereby it stimulates the production of sKlotho remains to be elucidated. Further, it is conceivable that sKlotho represents an adaptation mechanism by which the body attenuates increased IGF-1 actions. It has been found that sKlotho can inhibit the activation of the IGF-1 receptor (IGF-1R) in a dose-dependent manner [19, 33]. Subsequently, sKlotho-induced IGF-1R resistance is achieved by suppressing either the ligand-stimulated autophosphorylation and the activation of signaling events downstream of the IGF-1R [19, 34]. To the extent that sKlotho expression dampens IGF-1 actions in acromegaly patients, it might be a more reliable parameter for QoL than serum IGF-1, but this remains to be further studied.

A second finding of our study is that after switching to PAS-LAR therapy a significant improvement in QoL was observed without a significant change in IGF-1 levels. Although in some individuals (i.e., in the PAS-LAR monotherapy group), IGF-1 levels markedly decreased during PAS-LAR treatment, in most patients, IGF-1 levels did not decrease significantly. In contrast, during PAS-LAR treatment a significant increase in IGF-1 levels was observed in the combination therapy group compared with baseline. In addition, normalization of IGF-1 levels during PAS-LAR treatment was not achieved by all patients, while at baseline of the study IGF-1 concentrations within the age-adjusted normal range had been measured during combination treatment of first-generation SRL and PEGV. Our results confirm that relative lower or normalized levels of serum IGF-1 do not necessarily have consequences for a change in QoL [7–11]. This observation challenges the current strategy of using PAS-LAR as second or third-line monotherapy when IGF-1 levels have failed to normalize during first-generation SRL monotherapy, while a clinically relevant tumor remnant is still visible imaging and/or there is evidence for tumor growth [35].

In both groups we observed a significant decrease in GH levels during PAS-LAR treatment in comparison with baseline [1, 2]. We have previously assumed that this is related to the reduction or discontinuation of the PEGV dose [2]. This assumption is supported by the observed significantly lower GH levels in the PAS-LAR monotherapy group in comparison with the combination therapy group during follow-up [2]. In addition, the decreased GH levels may have had an impact on disease activity as well.

A third finding of our study is that most changes in QoL, which are possibly related to GH secretion, did not differ between patients using PAS-LAR alone and in combination with PEGV apart from one exception: a significant improvement in fatigue symptoms only presented in the combination group. However, quantitative changes in this subdimension did not differ significantly between the groups.

Soluble Klotho may be both an indicator of disease activity as well as a marker of QoL. Soluble Klotho levels at follow-up did not differ significantly between the two groups, possibly explaining why QoL did not differ significantly between the two groups. Moreover, total and age-dependent serum IGF-1 levels were significantly higher in patients treated with combination therapy compared with patients treated with monotherapy patients, possibly explaining why GH-dependent parameters of QoL did not differ between the two groups either. Unfortunately, the study was not powered to assess differences in QoL between monotherapy and combination therapy. In addition, the duration of follow-up of our study was relatively short, and some GH-mediated effects may become manifest only after long-term co-administration of PEGV therapy. Therefore, it is possible that studies in larger populations with a longer follow-up may show a difference in improvement in QoL between the two groups. Nevertheless, we did observe improvements in QoL in the total cohort that correlated well with other GH-dependent parameters such as headache, soft tissue swelling, and the AcroQoL physical subdimension, suggesting that in these patients, integrated GH action remains elevated despite normal IGF-1 levels.

It should be stressed that our study has several limitations. First of all, patients participating in the study could have a more proactive attitude, which helped them to achieve improvements in QoL in comparison with the general acromegaly population (i.e., selection bias). We should mention here that most patients remained biochemically controlled after switching to PAS-LAR therapy and correlations with IGF-1 levels were limited only for normal range IGF-1 levels. Secondly, general well-being might have increased in response to a reduction in the number of injections of PEGV that the patients received during the study in comparison with their previous treatment regimen. However, this may not account for differences in the change of QoL between both treatment groups. Lastly, participants were required to visit the hospital more frequently, which may influence improvements in QoL. All of these factors might have affected the patients’ perception of QoL.

What further complicates the interpretation of our results is the precarious understanding of sKlotho in the clinic. Although the sKlotho assay provides a reproducible method for quantitatively assessing sKlotho, it currently does not have a clear place in the clinical routine. An important step toward the implementation of measuring serum sKlotho in clinical routine is to run the assays on an automated system. Moreover, introduction of the standardization of sKlotho assays should be implemented and normal ranges of sKlotho in healthy controls should be developed.

Current consensus statements mainly aim at normalizing IGF-1 to the age-adjusted normal range [35], while more or less neglecting any signs and symptoms still present after IGF-1 (and GH) normalization. However, from a patient’s perspective, normalization of serum IGF-1 levels may be insufficient to treat his or her symptoms and signs. Therefore, we should include improvement of QoL as a treatment goal on top of the normalization of biochemical markers.

In conclusion, we observed significant improvements in QoL in biochemically well-controlled acromegaly patients during first-generation SRLs in combination with PEGV after switching to PAS-LAR treatment. We also found that sKlotho showed a positive correlation with improvements in QoL. Surprisingly, improvements in QoL were not accompanied by a significant change in IGF-1 levels, which challenges the importance of serum IGF-1 as a reliable parameter to monitor QoL from a patient’s perspective. Soluble Klotho concentrations may be a new useful marker of QoL in acromegaly patients which should be further studied.

## Supplementary material

Fig.S1

Supplementary Figure Legend

## Abbreviations

AcroQoL: Acromegaly Quality of Life
IGF-1: Insulin-like growth factor 1
IGF-1R: IGF-1 receptor
MID: Minimally important difference
PAPE: Pasireotide LAR and Pegvisomant Study in Acromegaly
PAS-LAR: Pasireotide long-acting release
PASQ: Patient-Assessed Acromegaly Symptom Questionnaire
PEGV: Pegvisomant
sKlotho: Soluble α-Klotho
SRL: Somatostatin receptor ligand
QoL: Quality of life
ULN: Upper limit of normal

## References

[1] A. Muhammad, E.C. Coopmans, P. Delhanty, A.H.G. Dallenga, I.K. Haitsma, J. Janssen et al. Efficacy and safety of switching to pasireotide in acromegaly patients controlled with pegvisomant and somatostatin analogues: PAPE extension study. Eur. J. Endocrinol. **179**(5), 269–277 (2018) 10.1530/EJE-18-0353 30076159

[2] A. Muhammad, van der Lely A.J., P.J.D. Delhanty, A.H.G. Dallenga, I.K. Haitsma, J. Janssen et al. Efficacy and safety of switching to pasireotide in acromegaly patients controlled with pegvisomant and first-generation somatostatin analogues (PAPE study). J. Clin. Endocrinol. Metab. **103**(2), 586–595 (2017) 10.1210/jc.2017-02017 29155991

[3] Beauregard C, Truong U, Hardy J, Serri O (2003). Long-term outcome and mortality after transsphenoidal adenomectomy for acromegaly. Clin. Endocrinol..

[4] Holdaway IM, Rajasoorya CR, Gamble GD, Stewart AW (2003). Long-term treatment outcome in acromegaly. Growth Horm. IGF Res.

[5] Holdaway IM, Rajasoorya RC, Gamble GD (2004). Factors influencing mortality in acromegaly. J. Clin. Endocrinol. Metab..

[6] Swearingen B, Barker FG, Katznelson L, Biller BM, Grinspoon S, Klibanski A (1998). Long-term mortality after transsphenoidal surgery and adjunctive therapy for acromegaly. J. Clin. Endocrinol. Metab..

[7] Biermasz NR, Van Thiel SW, Pereira AM, Hoftijzer HC, Van Hemert AM, Smit JWA (2004). Decreased quality of life in patients with acromegaly despite long-term cure of growth hormone excess. J. Clin. Endocrinol. Metab..

[8] Hua S-C, Yan Y-H, Chang T-C (2006). Associations of remission status and lanreotide treatment with quality of life in patients with treated acromegaly. Eur. J. Endocrinol..

[9] Biermasz NR, Pereira AM, Smit JWA, Romijn JA, Roelfsema F (2005). Morbidity after long-term remission for acromegaly: persisting joint-related complaints cause reduced quality of life. J. Clin. Endocrinol. Metab..

[10] Bonapart IE, van Domburg R, ten Have SMTH, de Herder WW, Erdman RAM, Janssen JA (2005). The ‘bio-assay’quality of life might be a better marker of disease activity in acromegalic patients than serum total IGF-I concentrations. Eur. J. Endocrinol..

[11] Rowles SV, Prieto L, Badia X, Shalet SM, Webb SM, Trainer PJ (2005). Quality of life (QOL) in patients with acromegaly is severely impaired: use of a novel measure of QOL: acromegaly quality of life questionnaire. J. Clin. Endocrinol. Metab..

[12] Schmid C, Neidert MC, Tschopp O, Sze L, Bernays RL (2013). Growth hormone and Klotho. J. Endocrinol..

[13] Matsumura Y, Aizawa H, Shiraki-Iida T, Nagai R, Kuro-o M (1998). Nabeshima Y-i. Identification of the HumanKlothoGene and its two transcripts encoding membrane and SecretedKlothoProtein. Biochem. Biophys. Res. Commun..

[14] Sze L, Bernays RL, Zwimpfer C, Wiesli P, Brändle M, Schmid C (2012). Excessively high soluble Klotho in patients with acromegaly. J. Intern. Med..

[15] Neidert MC, Sze L, Zwimpfer C, Sarnthein J, Seifert B, Frei K (2013). Soluble α-klotho: a novel serum biomarker for the activity of GH-producing pituitary adenomas. Eur. J. Endocrinol..

[16] J. Schweizer, M. Haenelt, K. Schilbach, A.Giannetti, M. Bizzi, B. Rocha et al. OR32-2 Alpha Klotho as a Marker of Disease Activity in Acromegaly. J. Endocr. Soc. **3**(Suppl 1), OR32–2 (2019). 10.1210/js.2019-OR32-2

[17] G. Anand, R. Bernays, M. Neidert, L. Regli, L. Sze, O. Tschopp et al. Reduction in serum biomarkers of acromegaly post-surgery and post-pharmacotherapy: are insulin-like growth factor (IGF)-1 and soluble (s)Klotho levels decreased to a similar extent? Endocr. Abstr. **63** P1066 (2019).

[18] Kohler S, Tschopp O, Sze L, Neidert M, Bernays RL, Spanaus KS (2013). Monitoring for potential residual disease activity by serum insulin-like growth factor 1 and soluble Klotho in patients with acromegaly after pituitary surgery: is there an impact of the genomic deletion of exon 3 in the growth hormone receptor (d3-GHR) gene on “safe” GH cut-off values?. Gen. Comp. Endocrinol..

[19] Kurosu H, Yamamoto M, Clark JD, Pastor JV, Nandi A, Gurnani P (2005). Suppression of aging in mice by the hormone Klotho. Science.

[20] Wolf I, Levanon-Cohen S, Bose S, Ligumsky H, Sredni B, Kanety H (2008). Klotho: a tumor suppressor and a modulator of the IGF-1 and FGF pathways in human breast cancer. Oncogene.

[21] Château M-T, Araiz C, Descamps S, Galas S (2010). Klotho interferes with a novel FGF-signalling pathway and insulin/Igf-like signalling to improve longevity and stress resistance in Caenorhabditis elegans. Aging.

[22] Neggers SJ, Kopchick JJ, Jorgensen JO, van der Lely AJ (2011). Hypothesis: extra-hepatic acromegaly: a new paradigm?. Eur. J. Endocrinol..

[23] Neggers SJCMM, van Aken MO, de Herder WW, Feelders RA, Janssen JAMJL, Badia X (2008). Quality of life in acromegalic patients during long-term somatostatin analog treatment with and without pegvisomant. J. Clin. Endocrinol. Metab..

[24] Bidlingmaier M, Friedrich N, Emeny RT, Spranger J, Wolthers OD, Roswall J (2014). Reference intervals for insulin-like growth factor-1 (igf-i) from birth to senescence: results from a multicenter study using a new automated chemiluminescence IGF-I immunoassay conforming to recent international recommendations. J. Clin. Endocrinol. Metab..

[25] Manolopoulou J, Alami Y, Petersenn S, Schopohl J, Wu Z, Strasburger CJ (2012). Automated 22-kD growth hormone-specific assay without interference from pegvisomant. Clin. Chem..

[26] Yamazaki Y, Imura A, Urakawa I, Shimada T, Murakami J, Aono Y (2010). Establishment of sandwich ELISA for soluble alpha-Klotho measurement: Age-dependent change of soluble alpha-Klotho levels in healthy subjects. Biochem. Biophys. Res. Commun..

[27] Webb SM, Prieto L, Badia X, Albareda M, Catala M, Gaztambide S (2002). Acromegaly Quality of Life Questionnaire (ACROQOL) a new health-related quality of life questionnaire for patients with acromegaly: development and psychometric properties. Clin. Endocrinol..

[28] Norman GR, Sloan JA, Wyrwich KW (2003). Interpretation of changes in health-related quality of life: the remarkable universality of half a standard deviation. Med Care..

[29] Badia X, Webb SM, Prieto L, Lara N (2004). Acromegaly quality of life questionnaire (AcroQoL). Health Qual. Life Outcomes.

[30] Webb SM (2006). Quality of life in acromegaly. Neuroendocrinology.

[31] Trainer PJ, Drake WM, Katznelson L, Freda PU, Herman-Bonert V, van der Lely AJ (2000). Treatment of acromegaly with the growth hormone-receptor antagonist pegvisomant. N. Engl. J. Med.

[32] Varewijck AJ, van der Lely AJ, Neggers SJ, Lamberts SW, Hofland LJ, Janssen JA (2014). In active acromegaly, IGF1 bioactivity is related to soluble Klotho levels and quality of life. Endocr. Connect.

[33] Wang Y, Chen L, Huang G, He D, He J, Xu W (2013). Klotho sensitizes human lung cancer cell line to cisplatin via PI3k/Akt pathway. PLoS ONE.

[34] Bartke A (2006). Long-lived Klotho mice: new insights into the roles of IGF-1 and insulin in aging. Trends Endocrinol. Metab..

[35] Melmed S, Bronstein MD, Chanson P, Klibanski A, Casanueva FF, Wass JAH (2018). A consensus statement on acromegaly therapeutic outcomes. Nat. Rev. Endocrinol..

