# Supplementary figures and images for: Soluble Klotho: a possible predictor of quality of life in acromegaly patients
Source: Endocrine. 2020 Apr 24;69(1):165–74. doi: 10.1007/s12020-020-02306-4 (PMC7343750; doi:10.1007/s12020-020-02306-4)

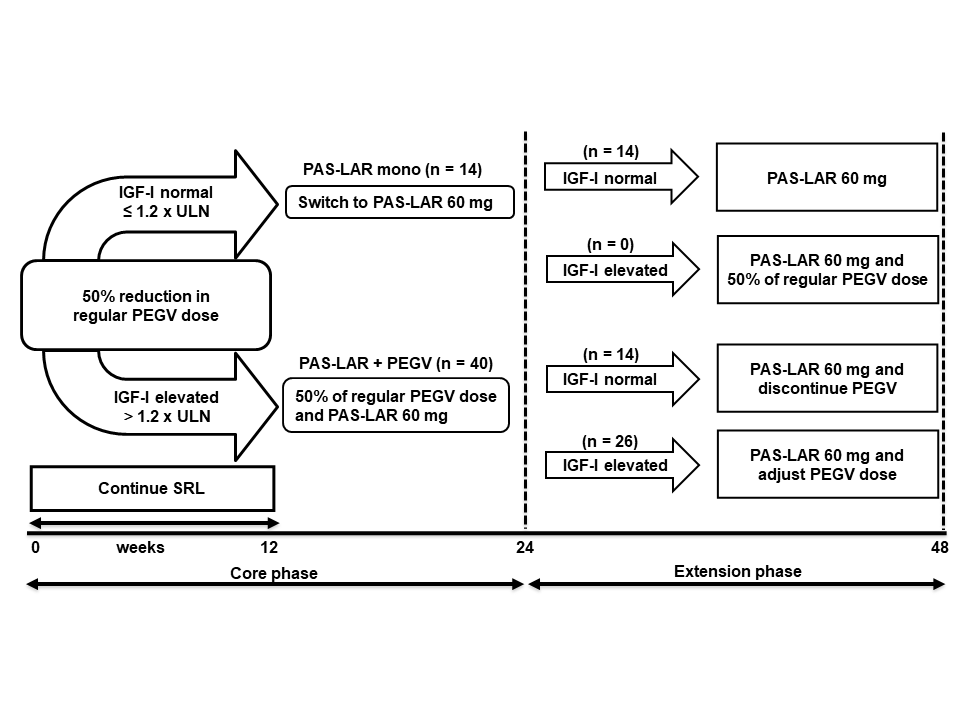

Supplement: Supplementary file 1 — Fig.S1 [file 12020_2020_2306_MOESM1_ESM.tif]
