# Supplementary material for: Soluble Klotho: a possible predictor of quality of life in acromegaly patients
Source: Endocrine. 2020 Apr 24;69(1):165–74. doi: 10.1007/s12020-020-02306-4 (PMC7343750; doi:10.1007/s12020-020-02306-4)
Supplement: Supplementary file 2 — Supplementary Figure Legend [file 12020_2020_2306_MOESM2_ESM.docx]

**Supplementary Figure Legend**

**Fig.S1** Study design of the PAPE study including the number of participants in each treatment group. All patients were previously treated with first-generation SRLs, followed by PEGV and first-generation SRL combination therapy. At baseline, the PEGV dose was reduced by 50% up to three months. When IGF-1 remained ≤1.2 × ULN after three months, patients were switched to PAS-LAR 60 mg monotherapy for three months. When IGF-1 was >1.2 × ULN, patients were switched to PAS-LAR 60 mg, and they continued with the 50% reduced PEGV dose for three months. During the extension phase until nine months of PAS-LAR treatment, the goal was to achieve IGF-1 normalization (IGF-1 ≤1.2× ULN) through protocol-based dose titration of PEGV and PAS-LAR. Insulin-like growth factor 1, IGF-1; PAS-LAR, pasireotide long-acting release; SRL, somatostatin receptor ligand; ULN, upper limit of normal.
